# Calcifying nested stromal-epithelial tumor of the liver: Report of two cases revealing novel *WT1* mutation and distinct epigenetic features

**DOI:** 10.1007/s00428-025-04281-5

**Published:** 2025-10-08

**Authors:** Andrea Strakova-Peterikova, Franco Fedeli, Boris Rychly, Jiri Soukup, Michael Michal, Petr Martinek, Marian Grendar, Elaheh Mosaieby, Nikola Ptakova, Maryna Slisarenko, Michal Michal, Kvetoslava Michalova

**Affiliations:** 1 Department of Pathology, Faculty of Medicine in Plzen, Charles University, Plzen, Czech Republic; 2 Bioptical Laboratory Ltd., Plzen, Czech Republic; 3 Cytopathos, Ltd., Bratislava, Slovakia; 4 Laboratorio Athena, Cesena, Italy; 5 Diagnostic Center of Pathology, Unilabs Slovakia Ltd., Bratislava, Slovakia; 6 Department of Pathology, Military University Hospital Prague, Prague, Czech Republic; 7 Department of Pathology, First Faculty of Medicine, Charles University, General University Hospital, Prague, Czech Republic; 8 Medical Laboratory CSD, Ltd., Kiev, Ukraine; 9 The Fingerland Department of Pathology, Charles University, Faculty of Medicine in Hradec Králové and University Hospital, Hradec Kralove, Czech Republic

**Keywords:** Liver, Calcifying nested epithelial and stromal tumor, CNSET, *CTNNB1*, *TERT*, *WT1*, Mutation, Genetics, Methylation analysis, SPN pancreas

## Abstract

Calcifying nested stromal-epithelial tumor (CNSET) is an extremely rare primary liver tumor of uncertain histogenesis that predominantly occurs in the pediatric age group and young adults. Knowledge regarding the molecular genetic profile of this entity remains limited, with only two molecular studies conducted to date, which identified pathogenic mutations in the *CTNNB1* gene and *TERT* promoter mutations in all analyzable cases. A more aggressive biological potential than previously reported has been only recently unveiled as well. To further advance the understanding of pathogenic mechanisms of CNSET and to investigate the distinctiveness of this rare entity, we analyzed two cases using immunohistochemistry, next-generation sequencing (NGS), and methylation profiling. The latter was employed to compare the epigenetic landscape of CNSET with that of clinicopathologically similar entities, such as hepatoblastoma and solid pseudopapillary neoplasm (SPN) of the pancreas. Both CNSET cases occurred in women (aged 24 and 23 years) and measured 24 cm and 16 cm in diameter, respectively. Both cases showed similar histological features, being composed of organoid nests of bland spindled to epithelioid cells embedded in myofibroblastic stroma. Both cases were immunohistochemically positive for CD56, WT1, and CAM5.2 and negative for hepatocellular and neuroendocrine markers. Case 2 showed aberrant nuclear expression of β-catenin, while in case 1, there was cytoplasmic positivity only. Using Illumina TruSight Oncology 500 NGS panel, case 1 revealed a pathogenic mutation in the *WT1* gene and a *TERT* promoter mutation, and case 2 had a *CTNNB1* mutation. DNA methylation analysis showed that CNSET forms a distinct cluster, separate from other reference entities. Follow-up in case 2 revealed a disease-free status 21 months after partial hepatectomy. This study showed that the molecular landscape of CNSET of the liver is characterized by *CTNNB1*, *TERT* promoter, and *WT1* gene mutations, with the latter representing a novel alteration. Similarly to *CTNNB1*, the *WT1* gene plays a significant role in the Wnt signaling pathway. Given the metastatic potential and chemotherapy resistance of CNSET, understanding its molecular background is important for potential alternative targeted treatment. Methylation profiling confirms CNSET as a distinct entity, separate from hepatoblastoma and SPN of the pancreas.

## Introduction

CNSET is an extremely rare primary liver tumor of uncertain histogenesis with a predilection for children and young adults, preferentially occurring in females [1–3]. Histologically, CNSET exhibits biphasic morphology, characterized by nests of relatively bland spindled to epithelioid cells embedded within a cellular myofibroblastic stroma. Immunohistochemically, most cases show positivity for WT1, CD56, broad-spectrum cytokeratins, and aberrant expression of β-catenin, along with negativity for hepatocellular and neuroendocrine markers [1, 3, 4]. The rarity of this tumor makes comprehensive large-scale analyses of its genomic background challenging. Only ten cases have been molecularly analyzed in two separate studies [1, 5]. Assman and colleagues performed mutational analysis of the *CTNNB1* gene using Sanger sequencing in two cases, revealing a *CTNNB1* mutation in exon 3 in both [5]. One of the tumors was unresectable and unresponsive to chemotherapy. Papke and colleagues were the first to conduct a comprehensive molecular study on eight cases of CNSET [1]. Using NGS, they identified *CTNNB1* and *TERT* promoter alterations in all analyzable cases. Beyond these genetic findings, the authors also unveiled a more aggressive biological behavior of CNSET than previously reported, as most patients in their series (four out of seven with available follow-up) developed distant metastases and were mostly unresponsive to chemotherapy. Aggressive cases tended to exhibit increased mitotic activity and/or lymphovascular invasion but were otherwise histologically indistinguishable from their indolent counterparts [1, 3].

The aim of this study was to advance our understanding of the molecular features of this exceedingly rare tumor by analyzing two cases of CNSET using comprehensive molecular genetic methods and to perform methylation analysis to investigate the relationship between CNSET and other potential mimics.

## Materials and methods

Two cases of hepatic CNSET were identified in our files. Three blocks were available for case 1 and two blocks for case 2. Both cases were immunohistochemically stained using a Ventana BenchMark ULTRA (Ventana Medical System, Inc., Tucson, AZ, USA) for CD56 (123C3, Ventana, RTU), WT-1 (6F-H2, Ventana, RTU), β-catenin (14, Ventana, RTU), HepPar1 (OCH1E5, Ventana, RTU), Arginase 1 (SP156, Ventana, RTU), INSM1 (A8, Santa Cruz Biotech, Dallas, TX, USA, 1:1000), synaptophysin (SP11, Ventana, RTU), chromogranin (DAK-A3, Dako, 1:200), and CAM5.2 (CAM5.2, Ventana, RTU).

### DNA and RNA sequencing

DNA and RNA sequencing (capable of detecting both gene mutations and fusion transcripts) was performed using the commercially available TruSight Oncology 500 kit (Illumina Inc), as described previously [6].

### Methylation profiling

Briefly, genomic DNA was extracted from formalin-fixed paraffin-embedded tissue sections for each of the samples. Next, 250 ng of genomic DNA was subjected to bisulfite conversion and processed on the Illumina Infinium Methylation EPIC/850k platform with over 850,000 methylation sites according to the manufacturer’s instructions. Publicly available methylation profiles for reference samples of seven tumor groups (hepatoblastoma, hepatocellular carcinoma, synovial sarcoma, epithelioid hemangioendothelioma, desmoplastic small round blue cell tumor, solid pseudopapillary tumor of the pancreas) were downloaded from the GEO database [7]. Data processing and analysis were performed using R (RRID:SCR_001905), version 4.4.3, and BioConductor (RRID:SCR_006442), with key libraries including minfi (RRID:SCR_012830), Rtsne (RRID:SCR_016342), and uwot. IDAT files for selected samples with GSM identifiers were downloaded using the GEOquery (RRID:SCR_000146) library.

### Preprocessing and probe selection

IDAT files from Illumina 450 K and EPICv1 arrays were imported into R, merged, and normalized using the preprocessNoob function. The same preprocessing pipeline was applied to EPICv2 IDAT files. Probes common to all three platforms (450K, EPICv1, and EPICv2) were identified, and MethylSets were subset and combined into a single dataset. Probes with cross-reactivity [8] and sex-associated probes [9] were removed. Detection *p*-values were calculated, and probes with a *p*-value > 0.05 in at least 10% of samples were excluded.

### Dimensionality reduction and visualization

The 2000 most variable probes (based on variance) were selected for downstream analysis. Before clustering, the beta-value matrix of these top probes was standardized. t-distributed stochastic neighbor embedding (t-SNE) and uniform manifold approximation and projection for dimension reduction (UMAP) were used for clustering, and results were visualized alongside a heatmap of the beta-value matrix.

## Results

### Clinicopathological and molecular genetic features

#### Case 1

**Fig. 1:**
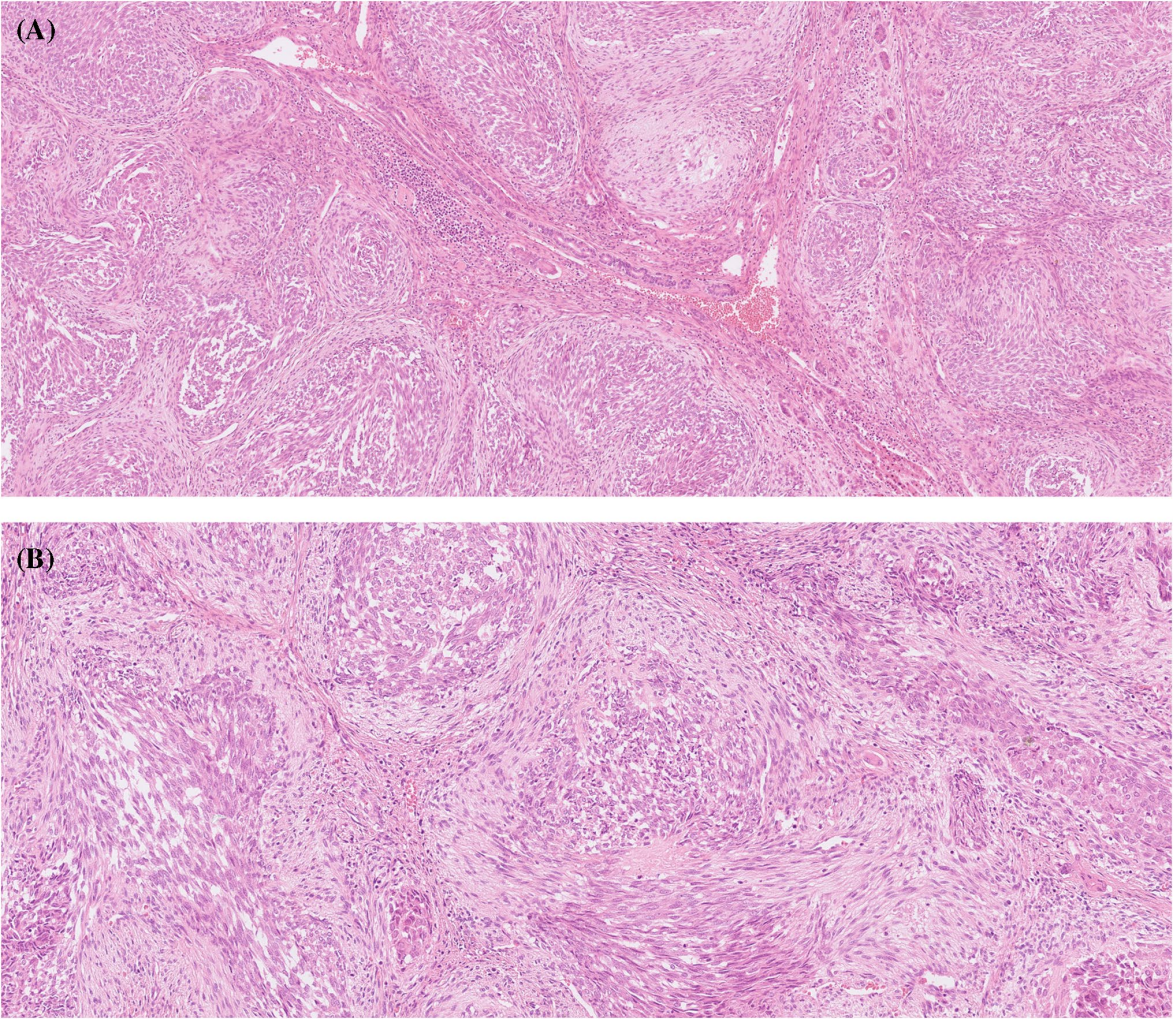
Histological features of case 1. The tumor consisted of biphasic nests of spindled and epithelioid cells encased by a variably prominent myofibroblastic proliferation. Occasionally, entrapped non-neoplastic bile ducts were present **A**. The tumor cells appeared bland, with moderate amounts of predominantly eosinophilic (less frequently pale) cytoplasm and bland ovoid, vesicular nuclei **B**

The tumor occurred in a 24-year-old woman with no further clinical or follow-up information available. Grossly, it was a solitary, well-circumscribed lesion measuring 24 cm in diameter, with elastic consistency and extensive centrally located ossified areas. Histologically, the tumor was composed of organoid nests of spindled to epithelioid cells with moderate amounts of predominantly eosinophilic (less often pale) cytoplasm and bland ovoid, vesicular nuclei. These nests were encased by a variably prominent myofibroblastic proliferation. Occasionally, entrapped non-neoplastic bile ducts were present (Fig. 1). Lymphovascular invasion and necrosis were not identified, and there was no mitotic activity (0 mitoses/10 high power fields (HPF)). There were signs of limited infiltration into the surrounding non-neoplastic liver parenchyma, but this could not be assessed extensively due to the limited amount of tissue available. Histologically, no calcifications were observed.

**Fig. 2:**
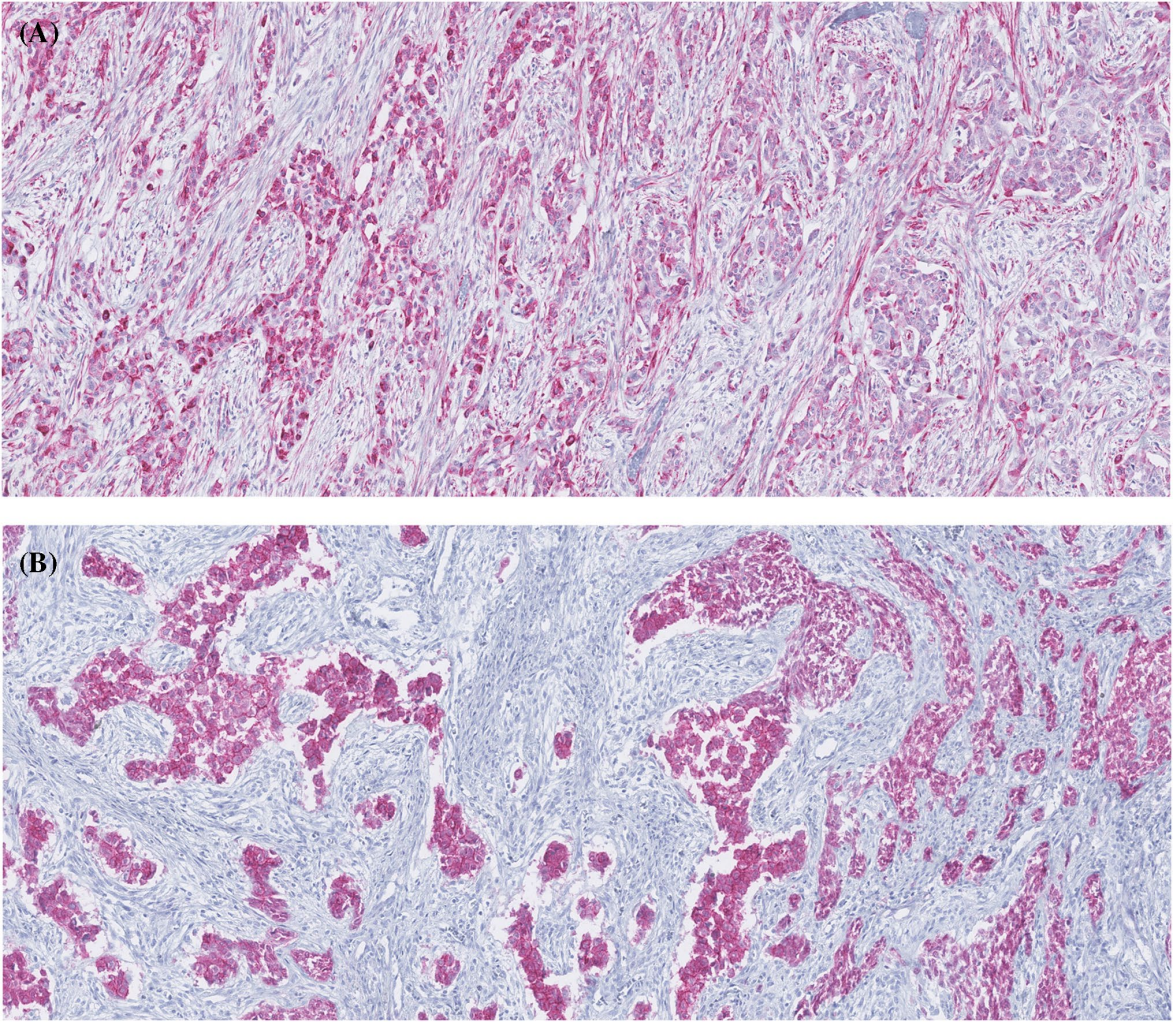
Immunohistochemically, case 1 stained positive for WT1 (focal nuclear expression) **A**, β-catenin showed only cytoplasmic positivity **B**

Immunohistochemically, the tumor stained positive for CD56, WT1 (focal nuclear expression), and CAM5.2 and was negative with hepatocellular markers (HepPar-1, arginase-1) and neuroendocrine markers (synaptophysin, chromogranin, INSM1). β-catenin showed only cytoplasmic positivity (Fig. 2). NGS detected a pathogenic *WT1* mutation (c.1484G > A p.(Arg495Gln); chr11:32410674; AF:10%) and a *TERT* promoter mutation (chr5:1295228C > T; AF: 9%), while *CTNNB1* gene mutations were absent. Additionally, mutations likely of germline origin in the genes *BMPR1A* and *PALB2* were identified. A non-tumor sample was not available to confirm the germline status of these variants. No fusion transcripts or clinically significant copy number alterations in the examined genes were identified.

The results of immunohistochemistry and NGS are summarized in Tables 1 and 2.

**Table 1:** Immunohistochemical features of two cases of CNSETs of the liver

	Case 1	Case 2
*WT1*	Cytoplasmic and focal nuclear positive	Cytoplasmic positive only
CD56	Positive	Positive
CAM5.2	Positive	Positive
β-Catenin	Cytoplasmic positive only	Aberrant nuclear and cytoplasmic expression
HepPar1	Negative	Negative
Arginase 1	Negative	Negative
synaptophysin	Negative	Negative
chromogranin	Negative	Negative
INSM1	Negative	Negative

**Table 2:** Molecular genetic features of two cases of CNSETs of the liver

Case 1	*TERT*	c.−124C > T		(alias C228T)	AF: 9%	NM_198253.3	chr5:1295228	hg19	Pathogenic/likely pathogenic mut
	*WT1*	c.1484G > A	p.(Arg495Gln)	(alias R495Q)	AF: 10%	NM_024426.6	chr11:32410674	hg19	Pathogenic/likely pathogenic mut
	*BMPR1A*	c.1456C > T	p.(Arg486Trp)	(alias R486W)	AF: 46%	NM_004329.3	chr10:88683246	hg19	Susp. germline mutation
	*PALB2*	c.509_510del	p.(Arg170IlefsTer14)	(alias R170I*X14)	AF: 45%	NM_024675.4	chr16:23647356	hg19	Susp. germline mutation
Case 2	*CTNNB1*	c.121A > G	p.(Thr41Ala)	(alias T41A)	AF: 20%	NM_001904.4	chr3:41266124	hg19	Pathogenic/likely pathogenic mut
	*CHEK2*	c.1175C > T	p.(Ala392Val)	(alias A392V)	AF: 49%	NM_007194.4	chr22:29091782	hg19	Susp. germline mutation
	*MRE11A*	c.1447C > T	p.(Arg483Ter)	(alias R483X)	AF: 60%	NM_005591.4	chr11:94192627	hg19	Susp. germline mutation

#### Case 2

The tumor presented as a solitary mass in a 23-year-old woman. The initial symptoms included dull abdominal pain, abdominal distension, and dyspepsia. On physical examination, a palpable mass was noted in the upper abdomen. Abdominal computed tomography revealed a well-circumscribed 16 cm hepatic mass. Imaging showed extensive vascular invasion involving the left and middle hepatic veins and the left portal pedicle. A preliminary diagnosis of fibrolamellar carcinoma or cholangiocarcinoma was considered, and the patient was scheduled for liver resection. Intraoperative ultrasonography confirmed the extent of vascular invasion, with no additional lesions identified. The patient underwent an extended left hepatectomy, including resection of the left and middle hepatic veins, to achieve complete removal of the tumor. The postoperative course was uneventful. The patient remained alive and disease-free for 21 months following surgery but was subsequently lost to follow-up.

Grossly, the lesion measured 16 cm, had well-defined margins, and was composed of yellow lobules with several granular foci.

**Fig. 3:**
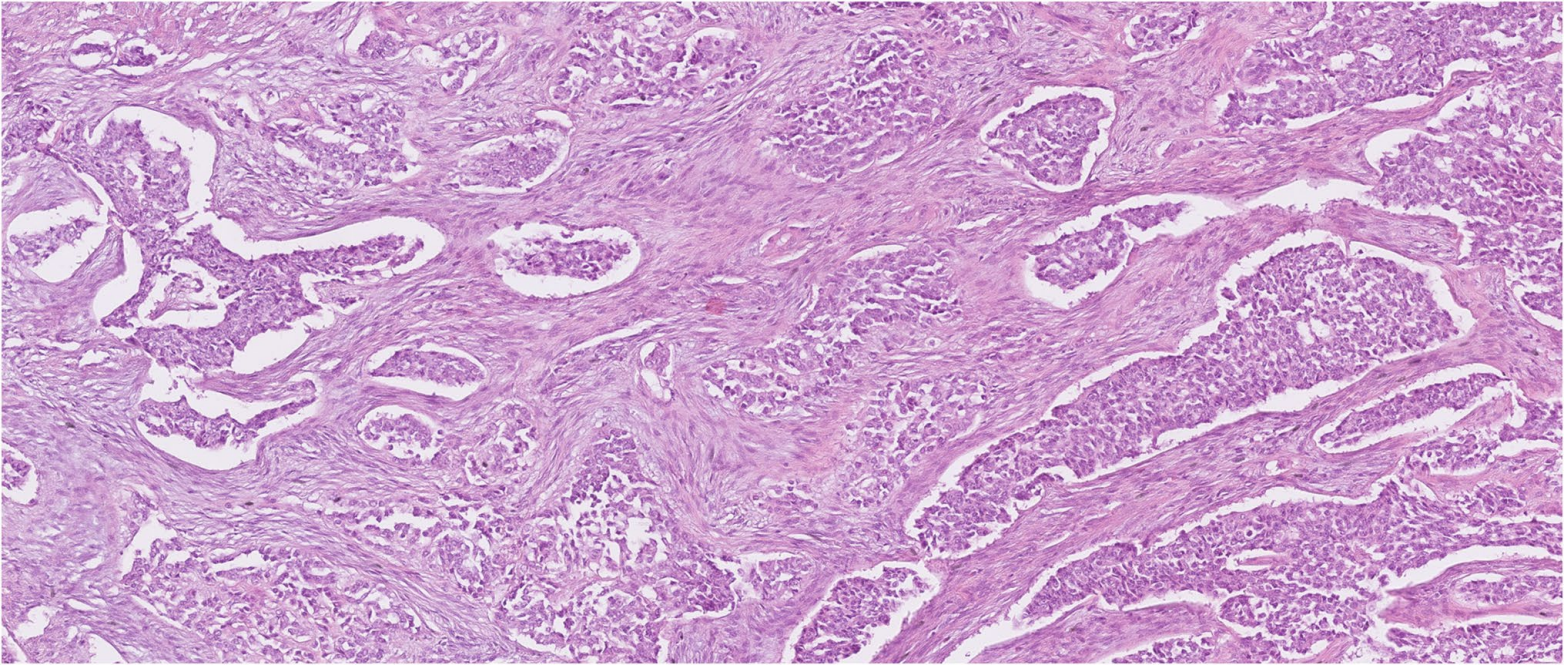
Histological features of case 2. It was a biphasic tumor largely similar to case 1. Retraction artifacts were occasionally noted between the nests and the surrounding myofibroblastic proliferation

Histologically, it was a biphasic tumor largely similar to case 1. Retraction artifacts were occasionally noted between the nests and the surrounding myofibroblastic proliferation (Fig. 3). Although extensive vascular invasion was observed radiologically, no evidence of lymphovascular invasion was identified histologically. This discrepancy was most likely attributable to the fact that the two available tissue blocks consisted exclusively of tumor tissue, without assessable non-neoplastic adjacent liver parenchyma. There was no evidence of necrosis, and mitotic activity was low (1 mitosis/10 HPFs). Due to limited tissue available, the relationship to the non-neoplastic liver could not be assessed. Microscopic examination revealed no calcifications.

**Fig. 4:**
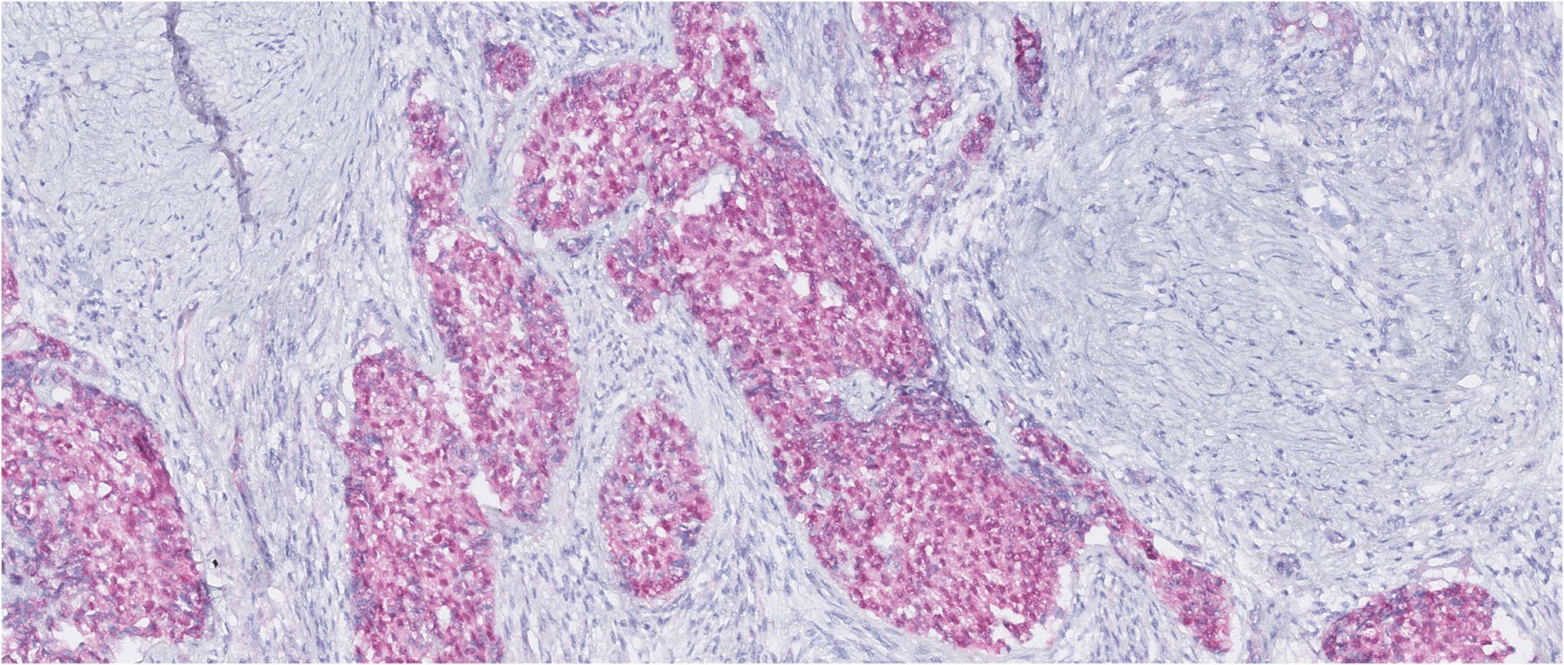
Immunohistochemically, case 2 showed aberrant nuclear and cytoplasmic expression of β-catenin

Immunohistochemically, the tumor showed aberrant nuclear and cytoplasmic expression of β-catenin (Fig. 4). WT1 showed only cytoplasmic positivity. The results for other immunohistochemical markers were identical as in case 1.

NGS detected a pathogenic mutation in the *CTNNB1* gene (c.121A > G p.Thr41Ala; chr3:41,266,124; AF:20%). Additional mutations, probably of germline origin, in the genes *CHEK2* and *MRE11A* were detected. A non-tumor sample was not available to confirm the germline status of these variants. No fusion transcripts or clinically significant numerical changes in the examined genes were identified. The results of immunohistochemistry and NGS are summarized in Table 1 and Table 2.

### Methylation profiling

**Fig. 5:**
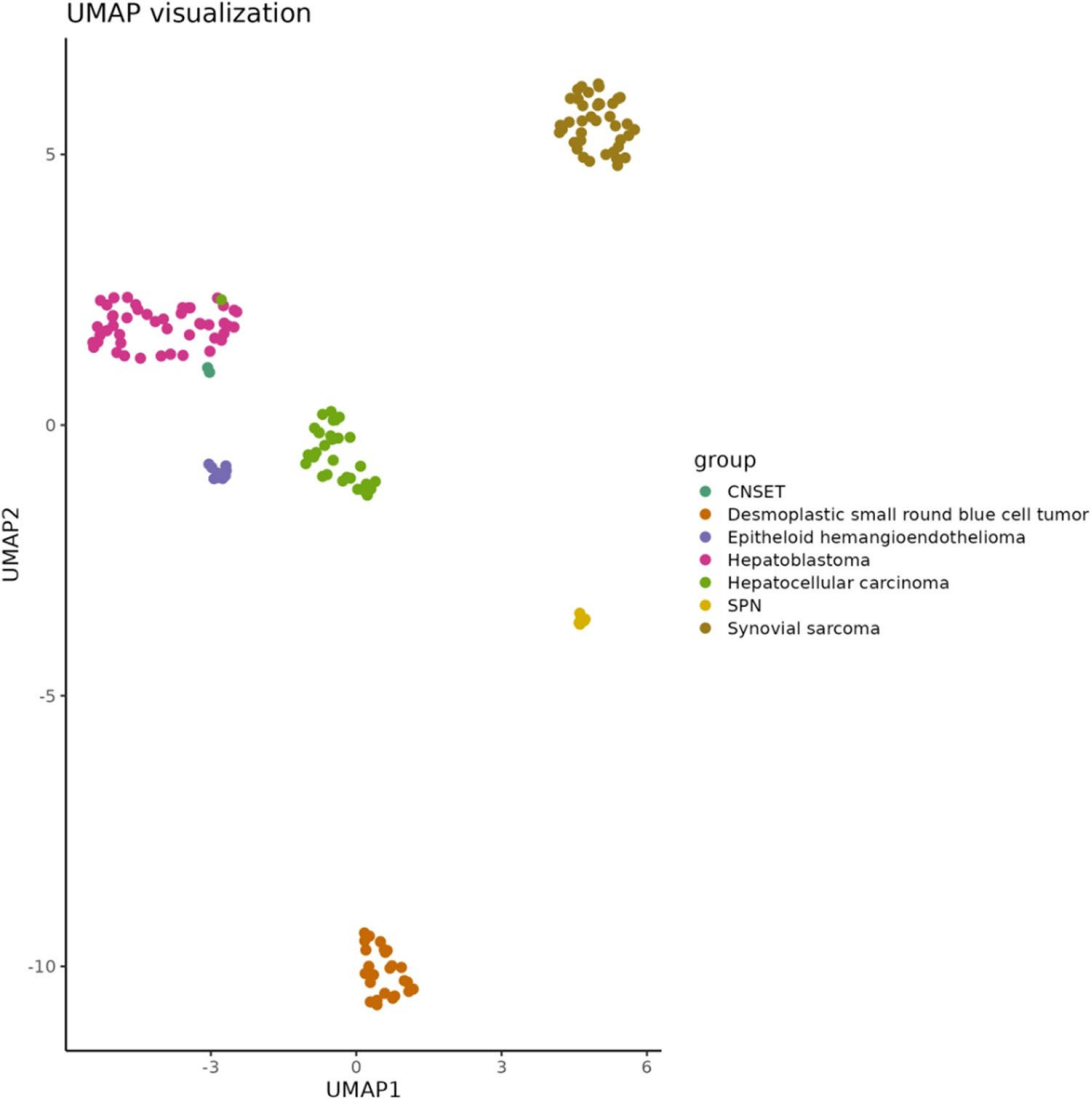
UMAP 2D projection of methylation profiles from patient samples representing seven tumor types: CNSET, desmoplastic small round blue cell tumor, epithelioid hemangioendothelioma, hepatoblastoma, hepatocellular carcinoma, SPN, and synovial sarcoma

The methylation profile effectively distinguished the seven patient groups (described above) as shown in the 2D UMAP representation in Fig. 5. The six SPN patients formed a tight, well-defined cluster, while the two CNSET patients also grouped distinctly. However, one hepatocellular carcinoma sample appeared as an outlier, positioned closer to the hepatoblastoma cases rather than clustering with the other hepatocellular carcinoma samples.

## Discussion

We report two cases of calcifying nested stromal-epithelial tumor (CNSET) of the liver that were analyzed using comprehensive molecular genetic methods, corroborating previous findings of *CTNNB1* and *TERT* promoter mutations in this tumor. Contrary to previous studies, which detected *CTNNB1* mutations in all CNSETs analyzed to date, one of our cases was *CTNNB1* wild-type and instead revealed *TERT* promoter and *WT1* mutations. This novel finding challenges the notion of *CTNNB1* mutations as a universal feature of CNSET, indicating that they are likely frequent but not absolutely required for the development of CNSET. It suggests that alternative molecular pathways, involving *TERT* and *WT1* mutations, can drive the formation of CNSET in the absence of *CTNNB1* alterations.

The *CTNNB1* gene encodes β-catenin, which is a well-established key player in both cell adhesion (as part of adherens junctions) and the Wnt signaling pathway [10, 11]. Mutations in *CTNNB1* lead to constitutive activation of the Wnt pathway, an early oncogenic event in many cancers [12–15]. This activation inhibits the β-catenin destruction complex, causing its accumulation and nuclear translocation, where it activates genes promoting cell growth and survival [16].

The *WT1* gene encodes a transcription factor essential for development and cellular regulation [17]. *WT1* mutations contribute to cancer development through diverse mechanisms beyond Wilms tumor, implicating it in a broad range of malignancies [18–20]. In liver cancer (hepatocellular carcinoma), *WT1* overexpression correlates with poor prognosis and chemoresistance [18]. *WT1* also complexly interacts with the Wnt signaling pathway, with studies suggesting both positive and negative regulatory relationships depending on the cancer type and cellular context [21–27]. The coordinated action of *WT1*, *WTX*, and *CTNNB1* within the Wnt pathway is critical for the development and survival of various malignancies [27].

*TERT* promoter mutations contribute to tumorigenesis by upregulating telomerase reverse transcriptase and stabilizing telomeres [28]. These somatic mutations are a common mechanism for telomerase activation in various human cancers, often linked to a more aggressive clinical course and poorer prognosis [29, 30]. Their frequent detection in CNSET strongly suggests a significant role for telomerase reactivation in the development and progression of this tumor, likely increasing *TERT* gene expression and enabling indefinite proliferation.

The presence of *TERT* promoter mutations and mutations in *CTNNB1* and *WT1* could potentially have synergistic or convergent effects that promote tumorigenesis and contribute to the unique histological and clinical features observed in CNSET. The characteristic nested morphology with spindled/epithelioid cells and a myofibroblastic component in CNSET of the liver suggests that altered epithelial-mesenchymal transition (EMT) may play a pathogenic role. EMT is a dynamic process in which epithelial cells acquire mesenchymal characteristics, enhancing their migratory and invasive properties and contributing to chemotherapy resistance [31–33]. Previous research on CNSET has supported this, demonstrating increased immunohistochemical expression of EMT-related markers such as c-Met, Twist, Snail, Slug, and vimentin, together with decreased E-cadherin expression [5]. This pattern is consistent with an impaired EMT process contributing to CNSET development. Furthermore, the genetic alterations implicated in CNSET are known to influence EMT. Mutations in *CTNNB1* are a significant driver of EMT. The Wnt/β-catenin signaling pathway itself is crucial in EMT induction and regulation, leading to the upregulation of key EMT-related transcription factors [34]. Additionally, *TERT* promoter mutations are linked to EMT, potentially through telomere-independent mechanisms or by interacting with signaling pathways that regulate this process [35]. Pan-cancer genomic analyses have revealed that a gene expression signature associated with *TERT* promoter mutations is significantly enriched for genes involved in EMT [36]. The *WT1* gene, which regulates numerous genes involved in cell growth, differentiation, and apoptosis [20], also likely influences EMT in a context-dependent manner, varying across different cancer types and stages [37]. These genetic alterations, combined with the observed expression in EMT markers, may collectively contribute to the biphasic epithelial/mesenchymal morphology characteristic of CNSET.

The lack of clinical and imaging information in case 1, along with the novel genetic findings, may raise questions about the accuracy of the diagnosis. Additionally, further immunohistochemical or other examinations could not be performed due to the consumption of tissue blocks. Since the morphology and immunoprofile of the tumor were identical to the molecularly typical case 2, we consider this sufficient evidence to regard case 1 as CNSET. Furthermore, the aforementioned coordinated action of WT1 and CTNNB1 within the Wnt pathway makes the novel WT1 mutation plausible [27].

Given the limited follow-up for our cases, we are unable to determine whether the detected gene mutations impact the biological behavior of CNSET. Nevertheless, due to the risk of malignant behavior of CNSET and its resistance to chemotherapy, understanding its molecular genetic background might be therapeutically valuable. The findings of this study provide a strong rationale for exploring targeted therapeutic strategies for CNSET, even if specific inhibitors are not yet clinically approved for this tumor type. The presence of *CTNNB1* and *WT1* mutations suggests that Wnt pathway inhibitors could be a promising target. These inhibitors are currently under investigation in other malignancies and are designed to prevent the nuclear translocation of β-catenin or its interaction with transcription factors [36, 38–40]. Similarly, the discovery of recurrent *TERT* promoter mutations—implicated in tumor proliferation and an aggressive clinical course in other cancers—offers the potential for the use of telomerase inhibitors. The translational value lies in recognizing that the molecular fingerprint of CNSET provides a clear basis for future research into specific, targeted therapies that could potentially overcome the tumor’s resistance to conventional chemotherapy.

The epigenomic characteristics of CNSET have not been previously explored. Given the uncertain histogenesis of CNSET, methylation profiling was employed to assess the distinctness of this neoplasm by comparing it to morphologically similar tumors, particularly hepatoblastoma and solid pseudopapillary neoplasm (SPN) of the pancreas. Hepatoblastoma, like CNSET, arises in young individuals and frequently expresses β-catenin [41]. In particular, the fetal subtype of hepatoblastoma is morphologically similar, exhibiting nests of primitive tumor cells. SPN was included in the analysis due to its overlapping morphological, immunophenotypic, and molecular features characterized by *CTNNB1* mutations [42–44]. The UMAP analysis revealed clear separation of tumors into distinct clusters, reflecting underlying methylation signatures associated with specific pathological subtypes with minimal overlap between groups, suggesting robust epigenetic differences and supporting the unique nature of CNSET.

In summary, this study expands the known genetic landscape of CNSET beyond *CTNNB1* and *TERT* promoter mutations to include *WT1* gene mutations. The finding of a genetically distinct subgroup of CNSET (those with *TERT* and *WT1* mutations but without *CTNNB1* mutations) underscores the potential molecular heterogeneity within this rare tumor entity. This heterogeneity could have implications for the clinical behavior of CNSET, including its metastatic potential and chemotherapy resistance. Understanding the interplay of these genetic alterations will be crucial for developing more targeted and effective therapeutic strategies. Including *TERT* and potentially *WT1* in diagnostic molecular panels for CNSET could improve diagnostic accuracy, especially in *CTNNB1* wild-type cases. Methylation analysis revealed that CNSET is epigenetically distinct from other neoplasms with overlapping clinicopathological and molecular features. The limitations of this study include its small sample size, which prevents broad generalizations, assessment of clinical impact due to limited follow-up, and definitive conclusions on the causality or synergistic effects of identified mutations.

## Data Availability

The datasets used and/or analyzed during the current study are available from the corresponding author upon reasonable request.
